# Meeting of the Liverpool Centre

**Published:** 1918-03-30

**Authors:** 


### THE COLLEGE OF NURSING.

### Meeting of the Liverpool Centre.

A MEETING in connection with the Liverpool Centre of the College of Nursing was held on Monday, March 25, in the Royal Infirmary. Miss E. M. Cummins (Matron of the Royal Infirmary) presided over an attendance which included the following matrons : Miss Worsley (Children's Infirmary), Hon. Secretary of the Centre; Miss Aspinall (Stanley Hospital), Miss Bagnall (Royal Southern Hospital), Miss Curbishley (Liverpool Parish Poor-Law Institution), Miss Drysdale (District Nursing Society), Miss Golding, Mrs. Roberts (Walton Poor-Law Institution), Miss Fraser (City Isolation Hospital, Grafton Street), and Miss Soden (Home for Incurables).

Miss Cummins said it was hoped to make the Liverpool Centre a model which other Centres might copy. They intended to make the Centre a place to which the majority working in the county would be able to go without unreasonable expenditure of time or money. They desired to elect a General Committee which would be thoroughly representative of all the hospitals and institutions.

The following Committees were elected : GENERAL COMMITTEE OF THE CENTRE :—*General Hospitals* : Miss Cummins (Royal Infirmary), Miss Aspinall (Stanley Hospital), Miss Bagnall (Royal Southern Hospital), and Miss Worsley (Children's Infirmary); *Poor-Law Hospitals* : Miss Scott (West Derby) and Miss Curbishley (Liverpool Parish); *District Nurses* : Miss Drysdale; *Private Nurses* : Miss Golding; *Maternity Hospital* : Miss Cauty; *Special Hospitals* : Miss Bramwell (Myrtle Street Eye Hospital) and Miss Lewin (Women's Hospital, Shaw Street); and Birkenhead, Miss McMillan.

EXECUTIVE COMMITTEE : Miss Cummins, Miss Worsley, Miss McMillan, and Miss Drysdale; Miss Worsley and Miss McMillan to be Joint Hon. Secretaries.

FINANCE COMMITTEE : Sir W. Scott Barrett, Alderman Crosthwaite, Mr. H. Wade Deacon, Mr. A. H. Maxwell, Mr. Thos. Woodsend, Mr. C. Cain, Mr. W. H. Oulton, Mr. H. P. Cleaver, and Dr. E. W. Hope (Medical Officer of Health for Liverpool)

Miss Cummins explained that they had chosen a Finance Committee composed of gentlemen because they were better at obtaining money than ladies.

A discussion ensued upon various matters connected with the future programme and organisation of the Centre. It was decided to have post-graduate lectures once a month between September and May each year.

The meeting, which was productive of various suggestions, transacted useful business of a formal character.

